# Multi-omics analyses of tumor-associated immune-infiltrating cells with the novel immune checkpoint protein tyrosine phosphatase 1B (PTP1B) in extracellular matrix of brain-lower-grade-glioma (LGG) and uveal-melanoma (UVM)

**DOI:** 10.3389/fimmu.2022.1053856

**Published:** 2022-12-22

**Authors:** Kun-Hao Bai, Ming-Jiao Zhu, Yi-Yang Zhang, Xue-Ping Li, Si-Liang Chen, Da-Wei Wang, Yu-Jun Dai

**Affiliations:** ^1^ State Key Laboratory of Oncology in South China, Sun Yat-sen University Cancer Center, Guangzhou, China; ^2^ Department of Endoscopy, Sun Yat-sen University Cancer Center, Guangzhou, China; ^3^ Department of Emergency, Peking University First Hospital, Beijing, China; ^4^ Department of Hematologic Oncology, Sun Yat-sen University Cancer Center, Guangzhou, Guangdong, China; ^5^ Department of Hematology, Peking University Shenzhen Hospital, Shenzhen, Guangdong, China; ^6^ National Research Center for Translational Medicine, Ruijin Hospital affiliated to Shanghai Jiao Tong University School of Medicine, Shanghai, China

**Keywords:** new immune checkpoint, MSI-1436, single-cell expression analysis, extracellular matrix, PTP1B

## Abstract

Immune checkpoint inhibitors represented by PD-1 have greatly changed the way cancer is treated. In addition to PD-1, new immune checkpoints are constantly excavated to better treat cancer. Recently, protein tyrosine phosphatase 1B (PTP1B) was identified as a new immune checkpoint and played a critical role in the treatment of tumors by inhibiting the proliferation and cytotoxicity of T cells induced by tumor antigen. To explore the targeting role of PTP1B in precision tumor therapy, we deeply analyzed the expression and prognosis of PTP1B in all tumors. Survival analysis results indicated that PTP1B was highly expressed in most tumor tissues and indicated poor prognosis in acute-myeloid-leukemia (LAML), brain-lower-grade-glioma (LGG), kidney-renal clear-cell-carcinoma (KIRC) and uveal-melanoma (UVM). The methylation status of PTP1B in these four tumors exhibited hypomethylation and mutation landscape showed that PTP1B had its specific characteristics in genomic instability and heterogeneity. The homologous recombination deficiency (HRD) and loss of heterozygosity (LOH) were positive related to PTP1B expression in liver-hepatocellular-carcinoma (LIHC) and kidney-chromophobe (KICH), while the immunescore and immune infiltration displayed a significant positive correlation with PTP1B expression in LGG and UVM. Drug sensitivity tests showed that the PTP1B inhibitor MSI-1436 had a sensitivity effect suppressing tumor cell viability and suggested it enhanced the efficacy of PD-1 inhibitors in cancers.

## Introduction

T cells are an important part of the body’s immune system, helping not only to clear invading pathogens, such as viruses, but also to kill cancer cells (1). Immune checkpoint therapy, represented by PD-1/PD-L1 inhibitors, is considered to have completely changed the cancer treatment landscape and has a good therapeutic effect on a variety of cancers, but not all patients respond, and with treatment progresses, drug resistance emerges (2, 3). Therefore, improving the effectiveness of PD-1 inhibitors or expanding their application scope has received high attention in clinical research.

PTP1B (encoded by PTPN1) belongs to the protein tyrosine phosphatase family (4). Together with protein tyrosine kinases, they maintain the balance of tyrosine protein phosphorylation, participates in cell signal transduction, and regulates cell growth, differentiation, metabolism, gene transcription and immunity (5). The study found that in the skeletal muscle and liver of PTP1B knockout mice, insulin receptor autophosphorylation was increased, sensitivity to insulin was improved, and weight gain was resistant (6–8). These studies clarify that PTP1B is a target for the treatment of type 2 diabetes and obesity and indicate that PTP1B inhibitors could effectively treat type 2 diabetes and obesity by improving insulin sensitivity (7).

It has been reported that PTP1B was closely related to the occurrence of multiple tumors and progress. In classical Hodgkin’s lymphoma, the PTPN1 splice variant was a positive adjustment factor for the JAK/STAT signal pathway. The single nucleotide polymorphisms (SNPs) of PTP1B were identified and could decrease overall risk in breast cancer. PTP1B activated the MAPK/ERK and PI3K/AKT pathways to accelerate the progression of glioma and indicated poor prognosis in glioma. Moreover, the phosphatase PTP1B was considered as a potential favorable marker of chemotherapy response in metastatic carcinoma. Recent study also indicated that PTP1B could be an intracellular checkpoint that limited antitumor immunity of T-cells and CAR-T therapy (9).

In this study, we explored the PTP1B expression in healthy and cancer tissues and evaluated its prognostic significance in cancers. Methylation and mutation profiling analysis were performed to interpret the differential expression of PTP1B in cancers. In addition, we used immune cell infiltration analysis to investigate the relationship between PTP1B and immune cells of tumor.

## Methods

### Sample preparation

The transcript level expression data of different types of cancers were obtained from the Genotype-Tissue Expression (GTEx) and The-Cancer-Genome-Atlas (TCGA) databases (10). We retrieved the relevant data of all types of cancers from TCGA projects especially including acute-myeloid-leukemia (TCGA-AML), kidney-chromophobe (TCGA-KICH), brain-lower-grade-glioma (TCGA-LGG), liver-hepatocellular-carcinoma (TCGA-LIHC) and uveal-melanoma (TCGA-UVM). Due to the lack of normal control of AML in TCGA, we used the data of normal bone marrow mononuclear cells in GTEx as the control. We used the log2(x+1) to normalize the data and perform differential gene expression by using “limma” package with a false discovery rate (FDR) < 0.05. The TCGA dataset also included the basic corresponding clinical information, such as age and gender of patients and the grade and stage level of diseases. Perl in R was applied to merge the corresponding data into a matrix workflow. The PTP1B expressions between cancer tissues and its corresponding adjacent tissues were compared by using t test. The Institutional Review Board of Sun Yat-Sen University Cancer Center approved this study. The human cancer tissues of newly diagnosed AML samples used in this study were with the code number GZR2020-152.

### Bulk and single-cell expression analysis

The databases including the Consensus and HPA datasets were used to compare the RNA expression level of PTP1B among different tissues and FANTOM5 dataset was used to compare the protein expression level of PTP1B among those tissues (11). In addition, the single-cell RNA-sequencing (scRNA-seq) of different tissues was analyzed (12, 13). First, quality control of cells used to scRNA-seq should be single suspension cells without pre-enrichment. Then, the cell number for sequencing data should more than 4,000 and the data amount must at least 20 million read counts. Final, the expression of PTP1B in tissues were visualized by UMAP.

### Subcellular localization

The immunofluorescence microscopy analysis of PTP1B was analyzed as previous described (14). PTP1B localization in A-431 (skin squamous carcinoma), U-2 OS (osteosarcoma) or U-251 MG (glioma) cells was showed in green and the nucleus was displayed by blue. The PTP1B antibody was obtained from Sigma-Aldrich (catalogue number: HPA012542, Rabbit, 0.1775 mg/ml) and Santa Cruz Biotechnology (catalogue number: CAB009329, sc-14021, Rabbit). The dilutions of the PTP1B antibodies used for immunofluorescence staining was 1:100.

### Quantitative real-time PCR (qRT-PCR)

PTP1B expression was measured by qRT-PCR. We used the assay (ESscience; QP002) according to instructions of the manufacturer and applied the formula 2^−ΔΔCt^ to analyze the relative expression data. Primers are listed PTPN1 Forward Primer: GCAGATCGACAAGTCCGGG; Reverse Primer: GCCACTCTACATGGGAAGTCAC. The primers were designed by Primerbank.

### Western blot analysis

The suspended cells obtained from the exponential phase of growth and centrifuged at 800 rpm for 5 min. Then, the cell pellets were resuspended by RIPA lysis (PHYGENE, fuzhou, China) buffer. The lysates underwent vortex and sonication in an ice water bath for 5 min, prior to be centrifuged at 12000 rpm at 4°C for 15 min. After protein quantification, protein was mixed with loading buffer, and boiled at 100°C for 10 min, followed by electrophoretic separation on a 10% SDS–PAGE, before transfer to PVDF membranes. The membrane was then treated with 5% skim milk powder for 2 h at room temperature to eliminate unspecific interaction, with subsequent overnight treatment with antibodies. Antibodies were purchased from Proteintech (anti-PTP1B, 11334-1-AP, 1:500), and Santa Cruz (anti-GAPDH, 1:1000). The results were visualized by enhanced chemiluminescence (ECL) western blot detection reagents (GBCBIO Technologies, Guangzhou, China).

### Reagent

PTP1B inhibitor MSI-1436 (Trodusquemine, GC32396) was obtained from GLPBIO. Tislelizumab, a humanized IgG4 anti-PD-1 monoclonal antibody, was sponsored by BeiGene.

### Cell viability assay

About 50,000 OCI-AML3 and MOLM13 cells (AML cell lines, from Da-Wei Wang laboratory), 293 (epithelial cells of human embryonic kidney), A375 (UVM cell lines, from Sun Yat-sen University Cancer Center) and U251MG cells (LGG cell lines, from Sun Yat-sen University Cancer Center) were cultured in 96-well plates with 90μl RPMI-1640 (Gibco, NY) contained 10% FBS (Biochrom AG, Germany). Add an appropriate amount of lymphocyte separating solution into the short and middle tube, take heparin anticoagulant bone marrow solution and fully mix it with the same amount of RPMI1640, slowly add it to the layered liquid surface along the tube wall with a dropper, and pay attention to maintaining a clear interface. Horizontal centrifugation 2000 rpm × 20 minutes. After centrifugation, the tube was divided into three layers, and there was a narrow band of white cloud layer dominated by mononuclear cells at the interface between the upper and middle layers. Use capillaries to insert into the cloud layer and absorb mononuclear cells. Put it into another short medium tube and add RPMI1640 1500rpm with a volume of more than 5 times × 10 minutes, wash the cells twice. After incubating 48 h, the cytotoxicity of MSI-1436 was measured by adding 10μl cell counting kit-8 (CCK-8, Japan) at 450 nm.

### Prognostic analysis of PTP1B

The prognostic indicators studied in this research contained the overall survival (OS), disease-specific-survival (DSS), progression-free-interval (PFI) and disease-free-interval (DFI). Forest-plot packages were applied to perform the univariate cox regression to analyze PTP1B prognosis. In addition, Kaplan-Meier analysis was applied to validate the important prognosis of PTP1B in pan-cancers. P < 0.05 was significant and the hazard ratio (HR) was calculated by 95% confidence interval.

### The immunohistochemistry pathology expression

The immunohistochemistry of PTP1B expression in patients with kidney-chromophobe (KICH), LIHC, LGG and UVM were analyzed by using the Human Pathology Atlas (15). Moreover, the expressions of PTP1B in tissues of different cancer types were analyzed by using protein databases such as HPA012542, CAB009329 and CAB015217.

### Mutation profiling analysis of PTP1B

The summary and substitution mutation distribution analysis of PTP1B was performed by Catalogue of Somatic Mutations in Cancer (COSMIC) (16). Different colors represent different mutation types. Moreover, the mutation profiling of PTP1B in LIHC, KICH, LGG, LAML and UVM was analyzed by sangerbox (http://sangerbox.com/Tool). Among them, a total of eight mutation types were included, such as missense mutation, in frame insertion, frame shift deletion, nonsense mutation, in frame deletion, frame shift insertion, splice site and translation start site.

### CpG methylation analysis of PTP1B

MEXPRESS database (https://github.com/akoch8/mexpress) was applied to analyze the CpG methylation status of PTP1B in different types of cancers (17). The methylated regions analyzed mainly contained CpG dinucleotide, CpG islands, gene body and transcript site and the clinical parameters could be selected based on our request. The statistical significance (p < 0.05) was calculated by Benjamin-Hochberg-adjusted p value and the correlation coefficients between PTP1B expression and methylation was analyzed by Pearson. * p < 0.05, ** p < 0.01, ** p < 0.001.

### Correlation coefficient analysis

The relationships among PTP1B expression and other methylation related genes (MLH1, MSH2, MSH6, PMS2 and EPCAM), immunoregulatory genes or immune checkpoint genes were analyzed by Pearson. In addition, the correlation of PTP1B expression with clinical features (including age, gender, stage and grade), genomic heterogeneity such as homologous recombination deficiency (HRD), loss of heterozygosity (LOH), ploidy, mutant-allele tumor heterogeneity (MATH), microsatellite instability (MSI) and tumor mutational burden (TMB) were calculated by Pearson. P-value <0.05 indicated significant.

### Immune cell infiltration and cell type specificity

The immune cell infiltration was analyzed by Estimate and TIMER databases (18). The Estimate database defined the pseudo-immune score included immunescore and stromalscore of samples as potential biomarkers of immune infiltration. The correlation coefficients of immunescore or stromalscore with PTP1B expression were analyzed by pearson and the results were visualized by ggplot2. TIMER datasets provided a validation on the correlation of immune cells with PTP1B expression. In addition, PTP1B expression in different types of blood cell lineages, such as T-cells, granulocytes, NK-cells, dendritic cells, B-cells, monocytes, progenitors and total peripheral-blood-mononuclear-cell (PBMC), were analyzed by HPA and Monaco datasets.

### Gene-set-enrichment analysis

Functional analysis on PTP1B expression was performed by using gene-set-enrichment-analysis (GSEA). Cluster profiler package was utilized to analyze the differential expressed genes in R software. These significant involved pathways associated with PTP1B expression enriched in KEGG pathways were showed in **Table S1**.

## Result

### Single-cell RNA expression level of PTP1B in tissues

**Figure 1:**
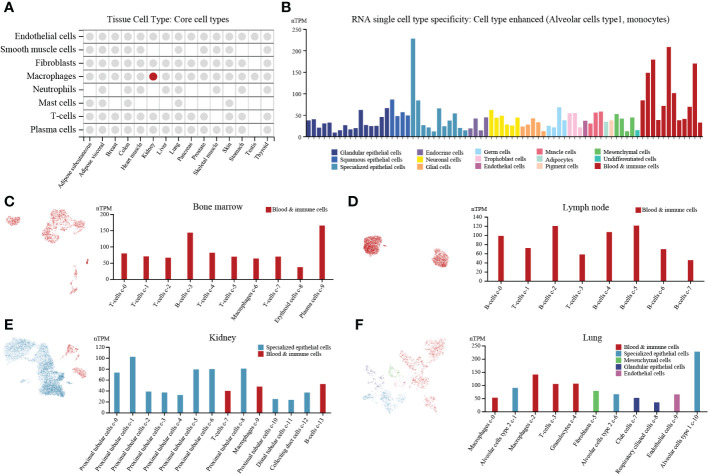
PTP1B expression in normal tissues/organs at single cell level. **(A)** PTP1B RNA expression in core cell types of some tissue cell types. **(B)** The cell type of PTP1B RNA single cell type specificity. The Alveolar cells type1 and monocytes showed enhanced. **(C-F)** PTP1B expression in all cells at single cell level. Single cell analysis of PTP1B expression in bone marrow **(C)**, lymph node **(D)**, kidney **(E)** and lung **(F)**.

PTP1B is an important molecule in the body’s immune regulation (4). First, we explored expression of PTP1B in different types of normal tissues and found that PTP1B was most highly expressed in respiratory system and bone marrow & lymphoid, followed by kidney & urinary bladder, gastrointestinal tract, proximal digestive tract and female tissues (**Figure S1A**). The PTP1B RNA expression was verified in other databases. The Consensus, HPA and GTEx databases suggested that PTP1B RNA tissue specificity was enriched in bone marrow, lung, lymph node, tonsil, parathyroid gland, kidney, spleen and appendix (**Figure S1B**). FANTOM5 database demonstrated that PTP1B protein expression was also higher expressed in caudate, lung, duodenum, small intestine, colon, kidney, fallopian tube, placenta, skin, appendix, spleen, lymph node and tonsil (**Figure S1C**). To explore PTP1B expression in tissue cell types, we analyzed the relevant data of core cell types, including endothelial cells, smooth muscle cells, fibroblasts, macrophages, neutrophils, mast cells, T-cells and plasma cells, and found that the macrophages in kidney was the cells most closely related to PTP1B expression (**Figure 1A**). The RNA single cell type specificity of PTP1B showed that the cell types were more enhanced in alveolar cells type1 and monocytes (**Figure 1B**). Next, we detailly analyzed subpopulation expression of PTP1B in these important association tissues by single-cell sequencing. The results indicated that immune cells had a relatively high PTP1B expression, especially in B-cells and plasma cells of bone marrow and lymph node (**Figures 1C, D**). In kidney and lung, PTP1B was more expressed in proximal tubular cells (kidney) and alveolar cells type1 and monocytes (lung), respectively (**Figures 1E, F**).

### PTP1B cellular localization and expression in cancers

**Figure 2:**
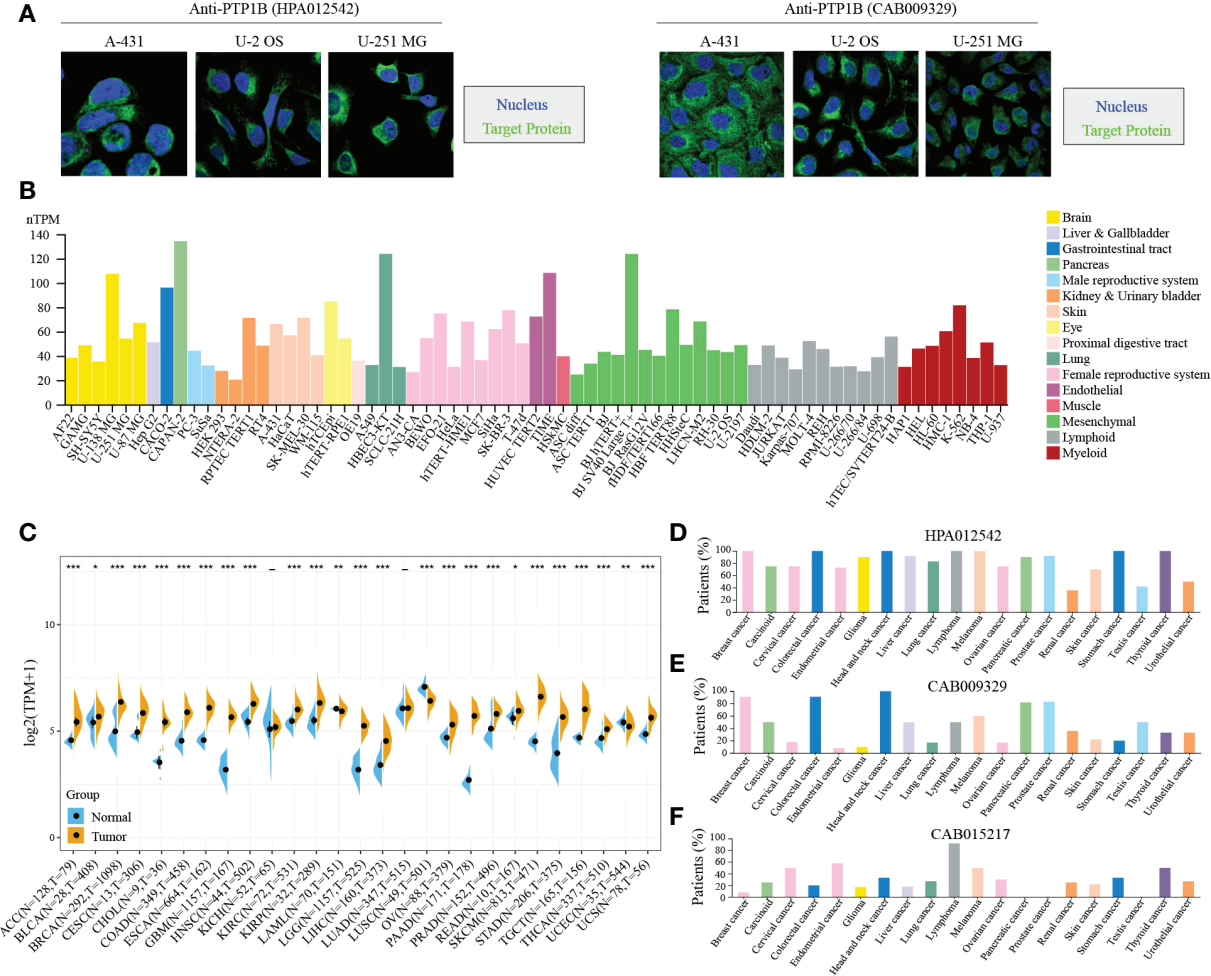
PTP1B in cancers. **(A)** PTP1B protein cellular localization in A-431, U-2 OS and U-251 MG cells by immunofluorescence. Blue presented nucleus; green suggested PTP1B. **(B)** PTP1B expression in cell lines of different cancer types from CCLE database. Different color corresponds to different tissues. **(C-F)** PTP1B expression in different types of cancers from TCGA and GETx database **(C)**, HPA012542 **(D)**, CAB009329 **(E)** and CAB015217 **(F)**. *p < 0.05; **p < 0.01; ***p < 0.001.

Next, we validated the cellular localization of PTP1B in tumor cells to evaluate its significance in pan-cancers. We chose three different cell lines to represent A-431 (carcinoma), U-2 OS (sarcoma) or U-251 MG (central system tumor). The immunofluorescence microscopy result indicated that PTP1B localized to cytoplasm in A-431, U-2 OS and U-251 MG (**Figure 2A**). Tumor cell line database results suggested that PTP1B is widely and highly expressed in all types of tumor cell lines (**Figure 2B**). Then, we further compared the PTP1B expression in all types of cancers and their adjacent tissues and the result indicated that PTP1B RNA expression was highly expressed in most cancer types except in acute myeloid leukemia (LAML), lung squamous cell carcinoma (LUSC) and uterine corpus endometrial carcinoma (UCEC) (**Figure 2C**). In addition, the PTP1B protein expression (HPA012542) was also highly expressed in most cancer cells and displayed strong cytoplasmic positivity with extra nuclear staining in several cases (**Figure 2D**). However, major cancers were weakly to moderately positive with PTP1B protein expression (CAB009329) (**Figure 2E**). With PTP1B protein expression (CAB015217), the lymphomas, melanomas, endometrial and cervical malignancies exhibited several strongly stained cases (**Figure 2F**).

### Prognosis of PTP1B in cancers

**Figure 3:**
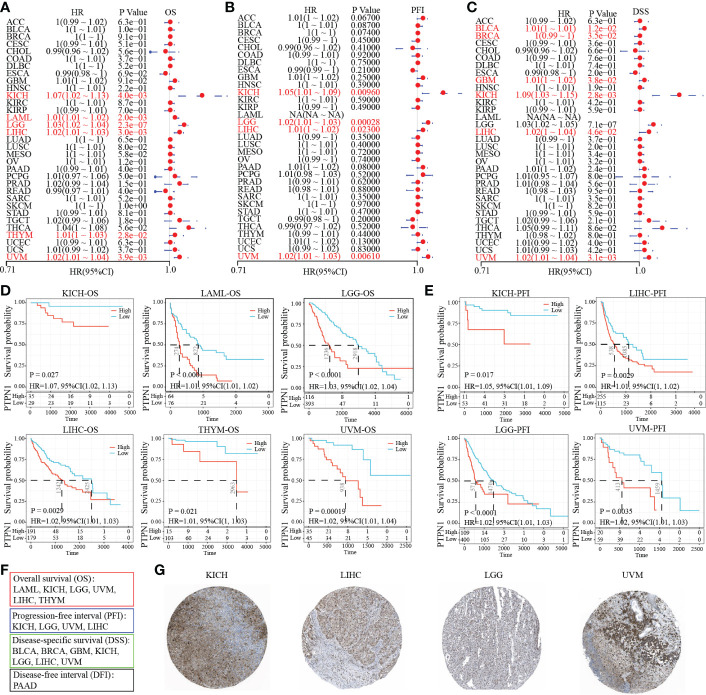
Prognostic value of PTP1B in different cancers. **(A-C)** The forest plot prognostic analysis of PTP1B on OS **(A)**, PFI **(B)** and DSS **(C)** in cancers. **(D)** Kaplan–Meier analysis of PTP1B on OS in KICH, LAML, LGG, LIHC, THYM and UVM. **(E)** Survival analysis of PTP1B on PFI in KICH, LGG, LIHC and UVM. P value and HR were showed in the bottom. **(F)** The summary of PTP1B survival related cancer types on OS, DFI, PFI and DSS. **(G)** Immunohistochemical expression of PTP1B in KICH, LGG, LIHC and UVM.

Further, we attempted to explore the prognosis of PTP1B in cancers. The forest plot prognostic analysis indicated that PTP1B expression could predict a poor survival rate of OS in KICH (HR=1.07), LAML (HR=1.01), LGG (HR=1.03), LIHC (HR=1.02), THYM (HR=1.01) and UVM (HR=1.02) (**Figure 3A**). In addition, PTP1B could also be poor prognosis markers in KICH, LGG, LIHC and UVM on PFI and in BLCA, BRCA, GBM, KICH, LIHC and UVM on DSS (**Figures 3B, C**). The OS, PFI and DSS rates of PTP1B expressed in those types of cancers were further validated by Kaplan–Meier analysis (**Figures 3D, E**). Finally, we integrated all survival index results as shown in **Figure 3F**. The prognosis of KICH, LGG, LIHC and UVM were most closely related to PTP1B expression. Immunohistochemistry further verified that PTP1B protein was highly expressed in these four tumor tissues (**Figure 3G**).

### Mutation and methylation landscape of PTP1B in cancers

**Figure 4:**
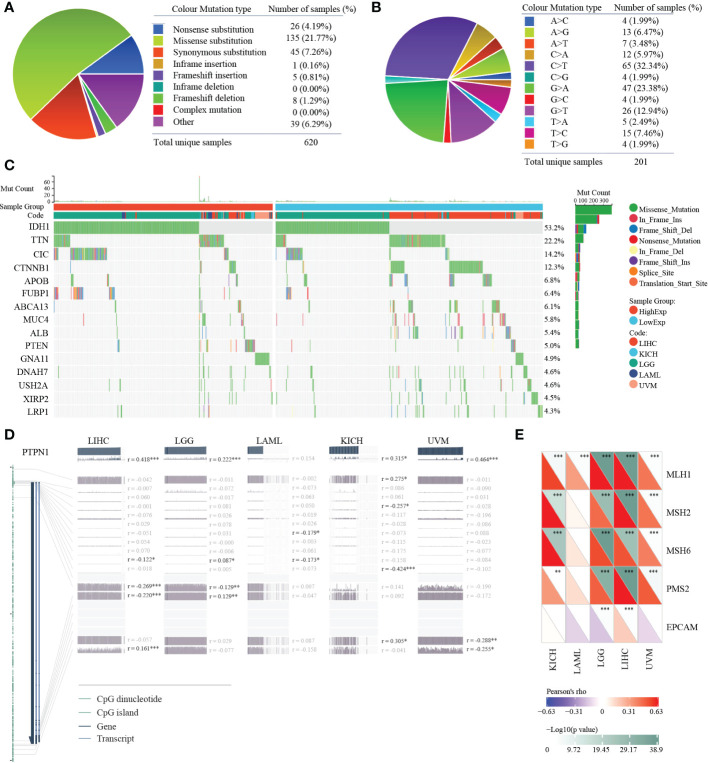
Mutation and methylation landscapes of PTP1B in cancers. **(A, B)** The summary **(A)** and substitution **(B)** mutation distribution analysis of PTP1B by COSMIC. **(C)** Mutation landscape of PTP1B in patients with LIHC, KIHC, LGG, LAML and UVM. **(D)** Methylation landscape of PTP1B in patients with LIHC, KIHC, LGG, LAML and UVM. **(E)** The correlation of PTP1B expression and methylation related genes (MLH1, MSH2, MSH6, PMS2 and EPCAM) in LIHC, KIHC, LGG, LAML and UVM. *p < 0.05; **p < 0.01; ***p < 0.001.

Gene mutation could affect its expression in tumors. Thus, we analyzed the mutation status of PTP1B in pan-cancers. The mutation landscape of PTP1B in cancers showed that the most occupied type was missense substitution (21.77%), followed by synonymous substitution (7.26%), then nonsense substitution (4.19%) (**Figure 4A**). The top three types of base mutations in the mutant gene were C>T (32.34%), G>A (23.38%) and G>T (12.94%) (**Figure 4B**). Among these five types of tumors, we divided them into high expression group and low expression group according to PTP1B expression. We analyzed and compared the differences in gene mutation profiles between the two groups in depth. These genes were frequently mutated in the four cancer types. Our results demonstrated that IDH1 was the most frequently mutated gene in the 5 types of tumors, with a total mutation rate of 53.2%. In the high expression group, the mutation frequency of IDH1 was exceeded 50%, which was significantly higher than that in the low expression group. Except for IDH1, CIC, FUBP1, PTEN and GNA11 had higher frequencies of mutations in the high expression group. while other genes did the exact opposite, such as TTN, CTNNB1, APOB, ABCA13, MUC4, ALB, DNAH7, USH2A, XIRP2 and LRP1 (**Figure 4C**). Next, we explored the methylation status of PTP1B among these types of cancers. These methylation peaks indicated that most of the CpG islands of PTP1B were in a state of hypomethylation, which led to the high expression of PTP1B (**Figure 4D**). In addition, we found that the demethylation genes, including MLH1, MSH2, MSH6, PMS2 and EPCAM, had a positive correlation with PTP1B (**Figure 4E**).

### Clinical features of PTP1B in cancers

**Figure 5:**
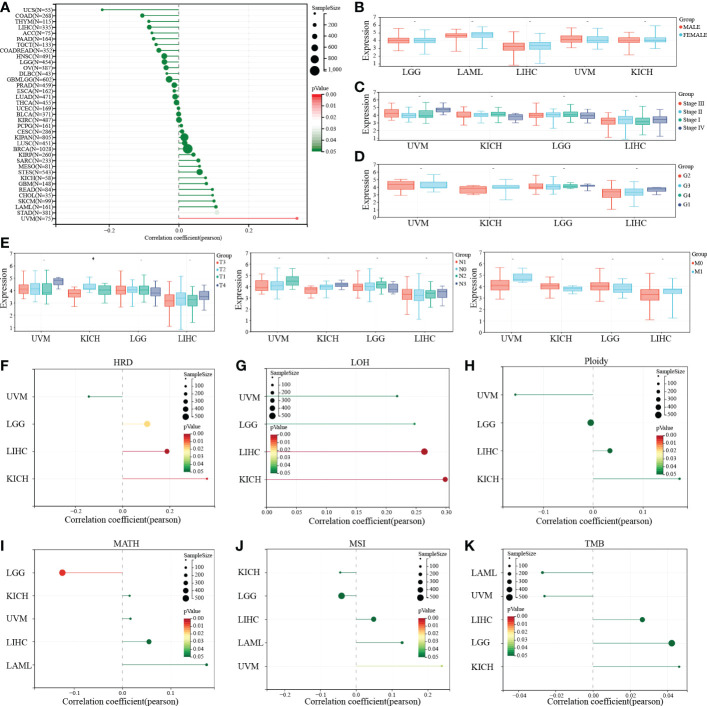
PTP1B and clinical characteristics in cancers. **(A-E)** The correlation of PTP1B expression and age **(A)**, gender **(B)**, stage **(C)**, grade **(D)** and TNM **(E)** in cancers. **(F)** Relationship between PTP1B expression and genomic heterogeneity, including HRD **(F)**, LOH **(G)**, ploidy **(H)**, MATH **(I)**, MSI **(J)** and TMB **(K)**. * p<0.05.

To further explore the effect of PTP1B expression on clinical symptoms of tumor patients. Pearson analysis was applied to analyze the correlation of PTP1B expression with basic clinical phenotypes. First, we found that age had no significant relationship with PTP1B expression in most cancers, except in UVM (**Figure 5A**). No significant correlation was found between PTP1B expression and sex, stage, grade or TNM in those above 4 types of cancers, neither (**Figures 5B–E**). Some recent reports indicated that tumor development is closely related to genomic heterogeneity, such as HRD, LOH, ploidy, MATH, MSI and TMB (**Figures 5F–K**). Our analysis suggested that PTP1B expression did not induce significant changes in genetic heterogeneity in these four cancer types. PTP1B expression was not associated with tumorigenesis on ploidy, MSI and TMI in four cancers and was positive related to LGG, LIHC and KICH on HRD and to LICH, KICH on LOH. While, in LGG MATH had a negative correlation with PTP1B expression.

### Immunscore and immune cells of PTP1B in extracellular matrix of cancers

**Figure 6:**
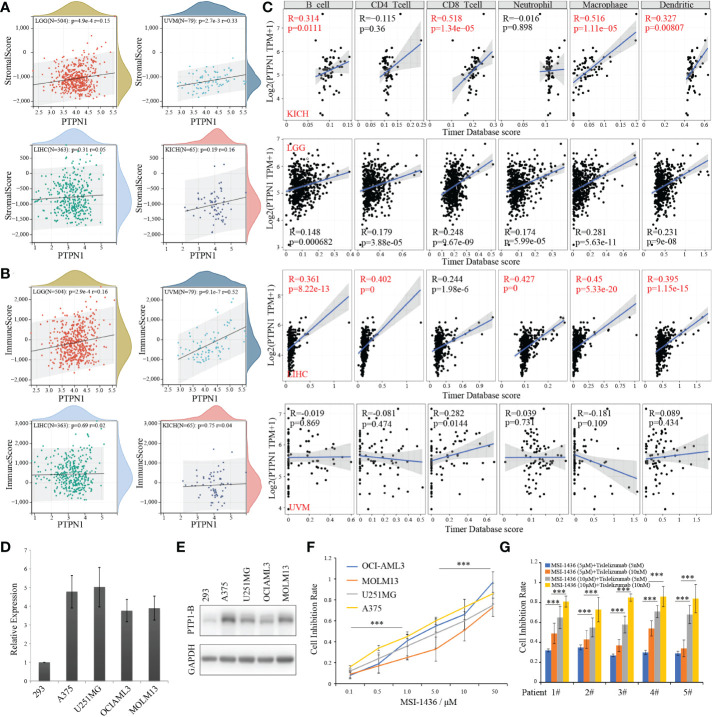
PTP1B, immune infiltration and CSCs index in cancers. **(A, B)** The correlation of PTP1B expression and stromalscore **(A)** or immunescore **(B)** in LGG, UVM, LIHC and KICH. The p value and correlation coefficient (r) were showed. **(C)** Immune infiltration of PTP1B in these four cancers. The correlation coefficient (r) was calculated by Pearson. **(D)** Relative PTP1B RNA expression validated in 293 and other cancer cell lines including A375, U251MG, OCI-AML3 and MOLM13 by qPCR. **(E)** Protein expression level of PTP1B in these cancer cell lines. **(F)** The cell inhibition rate of MSI-1436 (a PTP1B inhibitor) in different cancer cell lines with different doses. **(G)** Sensitivity effect of MSI-1436 and PD1 inhibitor (tislelizumab) in AML patient samples. ***p < 0.001.

As we known, PTP1B suppressed endogenous T cell-mediated antitumor immunity (9). To gain insight into the correlation of PTP1B expression with immune cells in these four cancers, we first calculated the immunescores of each patient according to the transcriptome data in these four types of tumors by ESTIMATE package. The stromalscores and immunescores displayed a significant positive correlation with PTP1B expression in LGG (p=4.9e-4 r=0.15; p=2.9e-4 r=0.16) and UVM (p=2.7e-3 r=0.33; p=9.1e-7 r=0.52), while they had no correlation with PTP1B expression in LIHC (p=0.31 r=0.05; p=0.69 r=0.02) and KIHC (p=0.19 r=0.16; p=0.75 r=0.04) (**Figures 6A, B**). Next, we validated the infiltrated immune cells in TIMER2 dataset. We defined the relationship (p<0.05 and r>0.3) as significance and found that PTP1B expression was related to B cells (r=0.314, p=0.0111), CD8-T cells (r=0.518, p=1.34e−05), macrophage (r=0.516, p=1.11e−05) and dendritic cells (r=0.327, p=0.00807) in KIHC and to B cells (r=0.361, p=8.22e-13), CD4-T cells (r=0.402, p=0), neutrophil (r=0.427, p=0), macrophage (r=0.45, p=5.33e-20) and dendritic cells (r=0.395, p=1.15e-15) in LIHC (**Figure 6C**). However, no significant immune cells were found had a closely relation to PTP1B expression in LGG and UVM (**Figure S2A**). In addition, we investigated the association of PTP1B expression and immunoregulatory gene expression in these four types of tumors. The results indicated that the expressions of genes involved in receptor and immunostimulatory and PTP1B were positively correlated and genes involved in chemokine, MHC and immunoinhibitory were negative correlated. We further examined the concordance of PTP1B expression and immune checkpoint genes expression. Almost all immune checkpoint genes are positively correlated with PTP1B at the expression level in KICH and LAML (**Figure S2B**).

### GSEA functional analysis of PTP1B

Next, we used GSEA analysis to explore the functional pathways related to PTP1B expression in cancers. Nearly 100 signaling pathways were enriched PTP1B-high express group. The results demonstrated that pathways involved in acute/chronic myeloid leukemia, non-small cell lung cancer, glioma, colorectal cancer, pancreatic cancer, renal cell carcinoma, prostate/bladder cancer and melanoma were more enriched in PTP1B-high express group. In addition, immune related pathways, such as chemokine signaling pathway, B/T cell receptor signaling pathway, natural killer cell mediated cytotoxicity and leukocyte transendothelial migration, were more aggregated in high expression groups (**Figure S3A**). Whereas, there was only two pathways enriched in PTP1B-low express group and the pathways were ribosome and oxidative phosphorylation (**Figure S3B**). This might further suggest the significance and strong relevance of cancer and immune infiltration and PTP1B.

### Sensitivity therapeutic effect of PTP1B inhibitor in tumors

Next, we explored the therapeutic effects of PTP1B inhibitor MSI-1436 on the viability in cancer cell lines. First, a relative high RNA and protein expression of PTP1B was validated in types of cancer cell lines (**Figures 6D, E**). In addition, we found that all these cell lines we used were sensitive to MSI-1436 with different IC50 after treatment for 2 days (**Figure 6F**). Our results indicated that the PTP1B expression had a closely relationship with the tumor-associated immune-infiltrating cells in extracellular matrix. Thus, we wonder whether PTP1B inhibitor has a synergistic effect with current immune checkpoint inhibitors such as PD1 or PDL1, TIM3, etc. We isolated mononuclear cancer cells from the bone marrow of AML patients to validate the effects of MSI-1436 *in vitro*. Consistent with the results from cell lines, patient cells treated with MSI-1436 were less viable, while a synergistic efficiency of MSI-1436 plus tislelizumab was found in primary cancer cells of AML patients (**Figure 6G**).

## Discussion

Immune checkpoint inhibitors, such as cytotoxic T lymphocyte-associated antigen-4 inhibitors, programmed cell death protein-1 inhibitors, and programmed cell death ligand-1 inhibitors, etc., can be used for the treatment of various malignant tumors, such as lung cancer, colorectal cancer, gastric cancer, melanoma, renal cell carcinoma, Hodgkin lymphoma, etc (19–22). In recent years, cellular immunotherapy, represented by CAR-T, transforms the T cells of cancer patients *in vitro* to make them recognize antigens on the surface of tumor cells, and then infuse these cells back into the patient to recognize and kill cancer cells (23, 24). Since 2017, the U.S. food and drug administration (FDA) has successively approved six CAR-T cell therapies for the treatment of blood cancers such as leukemia and lymphoma (24, 25). The successful application of CAR-T therapy has made many desperate people waiting for bone marrow matching (26). Hope has been rekindled, and it also marks the arrival of the era of cell therapy. However, after receiving CAR-T treatment, about 30%-40% of patients achieved durable remission, which also means that the treatment has limited effect on more than half of patients (24, 27). In addition, CAR-T is not effective for solid tumors, and there is currently no CAR-T therapy for solid tumors on the market (26). Here, we found PTP1B mainly expressed in important human organs and tissues, especially the respiratory system, gastrointestinal tract, reproductive system, urinary system and immune system, etc. Among them, macrophages were the most commonly affected tissue cell subtype. This further suggested that PTP1B had potential anti-tumor efficacy.

The phosphatase family can be divided into tyrosine phosphatases (such as SHP2, PTP1B, CD45, SHP1, PTPN2, PTPN14, PTPN22 and DUSPs) and serine/threonine phosphatases (such as PP2A, PP1, PP4, PP6 and PP2C) according to their amino acid substrates. SHP2 promoted BCR-ABL1-expressing myeloid and lymphoid neoplasia through MEK/Erk pathways (28). In addition, SHP2 was considered as a new therapeutic target for KNAS mutant non-small cell lung cancer and gastroesophageal cancer (29). Inhibition of SHP2 in breast cancer cells induced transformation of cancer cells into normal cells and inhibited tumor initiation and metastasis by blocking HER2 expression (28). High expression of SHP2 was related to the occurrence and development of pancreatic cancer (28). PTPN2 gene deletion increased the sensitivity of tumors to immunotherapy by regulating exhausted CD8+ T cells (30). Deletion or inactivation of PP2A could increase the infiltration of cytotoxic T cells in tumors and reshape the tumor immune microenvironment (31). In addition, the serine/threonine phosphatase PP2A allosteric activators had broad anti-tumor activity (32).

PTP1B was an important target for the treatment of metabolic diseases and played an important role in coordinating the regulation of energy metabolism and glucose homeostasis in the central nervous system (6). More and more studies reported that PTP1B play a very important role in development and progression of tumors. PTP1B promoted cell invasion by increasing EGFR protein expression through MYH9 in esophageal cancer cells (33). PTPN1 splice variants could up-regulated JAK/STAT activity in classic Hodgkin lymphoma cells, accelerated tumor cell proliferation, and reduced the toxicity effect of chemotherapy drugs such as gessabine and oroposide (34, 35). PTPN1 mutations were present in 3% of breast implant-associated lymphomas, and this mutation not only activated the JAK/STAT signaling pathway, but also caused loss of function in epigenetic regulatory regions (34). The significance of PTP1B was further evaluated in cancers. PTP1B was highly expressed in most tumor tissues and indicated poor prognosis in LAML, LGG, KIRC and UVM by Kaplan-Meier analysis.

In order to further explore the molecular mechanism of PTP1B regulation, we conducted in-depth analysis from the landscape of mutation and methylation profiles. Our data indicated that PTP1B was more common in tumors by missense substitution, of which cytosine to thymine mutation was the most common. In addition, like other genes, PTP1B was often accompanied by mutations in other genes (36). For example, PTP1B almost coexisted with IDH1 mutation in LGG and the most common mutation in LICH was TTN. IDH was a key enzyme in the tricarboxylic acid cycle, and IDH1, IDH2 gene mutation status was associated with the prognosis of tumor patients (37). IDH1, IDH2 gene was a potential tumor early diagnosis, prognosis evaluation and target therapeutic marker genes. TTN was a common driver gene for liver cancer (38). High TTN and lncRNA-TTN-AS1 expression were positively associated with poor overall survival in SKCM patients and could be considered as novel biomarkers and drug targets for SKCM patients (39). These results suggested that PTP1B as a synergistic molecule with other mutations participated in the whole process of tumorigenesis and development. In addition, the analysis of methylation profiling indicated that PTP1B was generally hypomethylated in these four types of tumors, which was closely related to the high expression of PTP1B in tumors.

Genomic heterogeneity analysis showed that the HRD and LOH had a positive correlation with PTP1B expression in LIHC and KICH. However, they were not related in UVM and LGG. Inconsistent with other solid tumors, AML was a hematopoietic malignancy, and there was no stage, grade, metastasis, HRD, LOH, ploidy of AML. Therefore, we lacked the relevant data in this part of AML. This suggested that among the four types of tumors, the mechanisms of PTP1B involved in tumor development and development were different. PTP1B played a key regulatory role in dendritic cell maturation and migration, and T cell activation. Using ESTIMATE to estimate tumor purity based on stromal and immune cell ratios correlated with tumor prognosis and was widely used in the study of various tumors. Our immune infiltration results demonstrated that PTP1B was positive related to stromalscore and immunescore in LGG and UVM. Moreover, PTP1B also increased immune cells infiltration, especially B cells, neutrophil, macrophage, dendritic cells in KICH and LIHC. These results indirectly suggested that PTP1B inhibitor might have a synergistic effect with tumor immunotherapy, especially in UVM and LGG. Functional analysis suggested that tumor pathways related to high expression of PTP1B were directly participated in the tumor development and immune-related pathways were also influenced by PTP1B in cancers.

The PTP1B inhibitor MSI-1436 effectively suppressed diet-induced obesity and reduced body fat content in mice (40). MSI-1436 is a selective, non-competitive inhibitor of PTP1B. In HepG2, MSI-1436 alone has no effect on IRβ phosphorylation, but in conjunction, MSI-1436 could significantly increase p-IRβ and STAT while treated with insulin (40). PTP1B was reported play an important role in endoplasmic reticulum stress (ERS) regulation of endothelium. The reduction of endothelial ERS by inhibiting PTP1B played endothelial protection in diabetes and cardiovascular diseases (41). At present, MSI-1436 has completed human safety trials and is safe and tolerated in humans (42). A query in clinicaltrials.gov found that PTP1B inhibitor was also undergoing many clinical trials. In mice, the PTP1B inhibitor MSI-1436 was showed to increase immune cell infiltration in tumors, inhibit tumor growth, and enhance the efficacy of PD-1 inhibitors. Tislelizumab was a humanized IgG4 anti-PD-1 monoclonal antibody. The mechanism of tislelizumab was combined with the PD-1 receptor on the surface of the T cells; the latter was an important immunosuppressive molecule, which could inhibit the activation of T cells and reduce the role of the immune system. However, no matter in these tumor cells or in primary tumor specimens, they did not include sufficient T cell participation reactions. There were T cells in the bone marrow of AML patients, which could cause immunotherapy. In addition, the normal T cells from individuals with solid tumors were not permitted in our center. Thus, we only verified the synergistic efficiency of MSI-1436 plus tislelizumab in cancer cells from AML patients instead of KICH, LGG, LIHC or UVM. Further, we are also trying to explore the synergy mechanism of MSI-1436 and tislelizumab. Whether MSI-1436 will induce tumor cell surface PDL1 protein expression and whether to regulate downstream related signal channels? Next, we are planning to perform experiment and a prospective control cohort clinical trial to verify this hypothesis in next work. As our results showed a high expression of PTP1B in normal tissue of the respiratory system, bone marrow and lymphoid tissue, chemotherapy and targeted drugs had different degree of adverse reactions. We speculated that PTP1B inhibitors also had several side effects including digestive tract discomfort, immune-related pneumonia, bone marrow suppression, endocrine disorders and cytokine storm. We will further observe the adverse reactions in our next prospective control cohort clinical trial.

## Data availability statement

The original contributions presented in the study are included in the article/**Supplementary Material**. Further inquiries can be directed to the corresponding author.

## Ethics statement

The studies involving human participants were reviewed and approved by The Institutional Review Board of Sun Yat-Sen University Cancer Center. The human cancer tissues of newly diagnosed acute myeloid leukemia (AML) samples used in this study were with the code number GZR2020-152. The patients/participants provided their written informed consent to participate in this study.

## Author contributions

K-HB and Y-JD designed the study. K-HB and YYZ performed the data analysis. X-PL, S-LC helped with the validation. K-HB and Y-JD wrote the manuscript. D-WW helped the revision. All authors contributed to the article and approved the submitted version.

## Supplementary material

Supplementary Table 1 GSEA report for high and low groups based on PTP1B expression

Supplementary Figure 1 PTP1B expression in normal tissues/organs. (A) RNA expression distribution of PTP1B in normal tissue/organs. Colors indicated different tissues/organs. (B, C) PTP1B RNA expression profiling in healthy tissues/organs from Consensus database (B) and FANTOM5 dataset (C).

Supplementary Figure 2 The relationships among PTP1B expression and immunoregulatory genes (A) or immune checkpoint genes (B) were analyzed by Pearson *p < 0.05.

Supplementary Figure 3 GSEA analysis of PTP1B in cancers. (A, B) Snapshot of enrichment results in phenotype PTP1B High-express group and Low-express group in cancers by GSEA.
