# Supplementary figures and images for: Multi-omics analyses of tumor-associated immune-infiltrating cells with the novel immune checkpoint protein tyrosine phosphatase 1B (PTP1B) in extracellular matrix of brain-lower-grade-glioma (LGG) and uveal-melanoma (UVM)
Source: Front Immunol. 2022 Dec 22;13:1053856. doi: 10.3389/fimmu.2022.1053856 (PMC9815505; doi:10.3389/fimmu.2022.1053856)

a

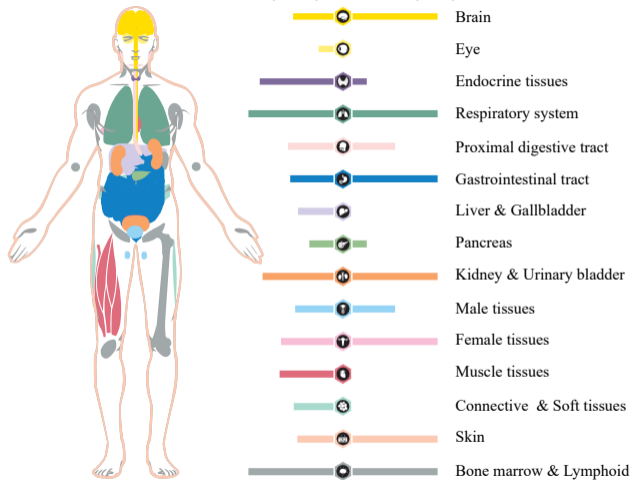

b

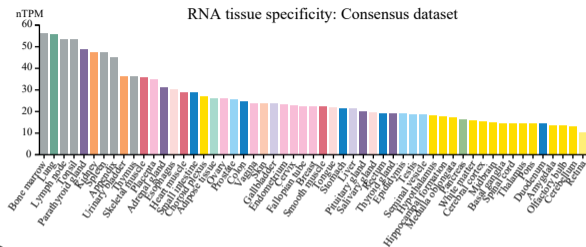

c

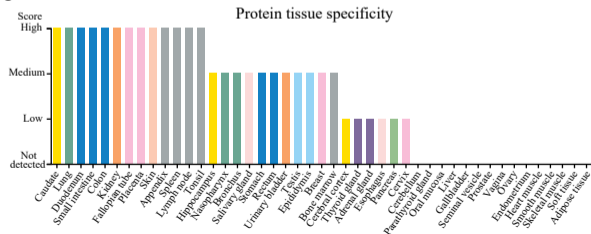

Supplement: Supplementary Figure 1 — PTP1B expression in normal tissues/organs. (A) RNA expression distribution of PTP1B in normal tissue/organs. Colors indicated different tissues/organs. (B, C) PTP1B RNA expression profiling in healthy tissues/organs from Consensus database (B) and FANTOM5 dataset (C). [file DataSheet_1.pdf]

a

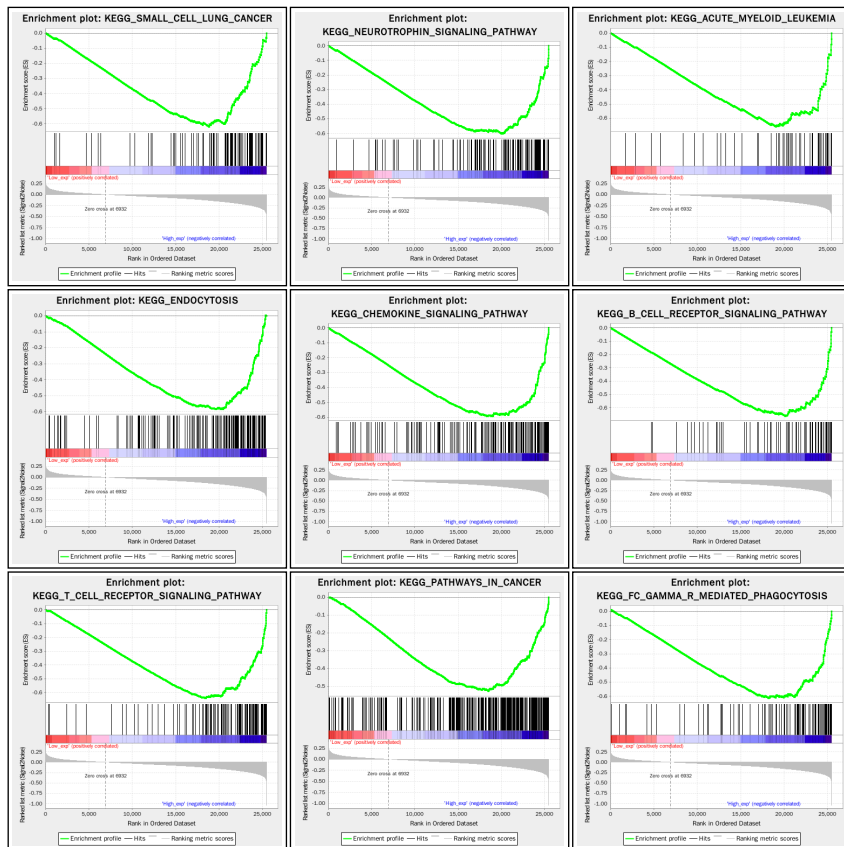

b

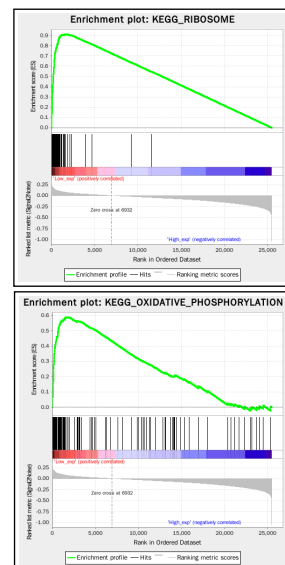

Supplement: Supplementary Figure 3 — GSEA analysis of PTP1B in cancers. (A, B) Snapshot of enrichment results in phenotype PTP1B High-express group and Low-express group in cancers by GSEA. [file DataSheet_3.pdf]
