# Supplementary material for: Multi-omics analyses of tumor-associated immune-infiltrating cells with the novel immune checkpoint protein tyrosine phosphatase 1B (PTP1B) in extracellular matrix of brain-lower-grade-glioma (LGG) and uveal-melanoma (UVM)
Source: Front Immunol. 2022 Dec 22;13:1053856. doi: 10.3389/fimmu.2022.1053856 (PMC9815505; doi:10.3389/fimmu.2022.1053856)
Supplement: Supplementary Figure 2 — The relationships among PTP1B expression and immunoregulatory genes (A) or immune checkpoint genes (B) were analyzed by Pearson *p < 0.05. [file DataSheet_2.pdf]

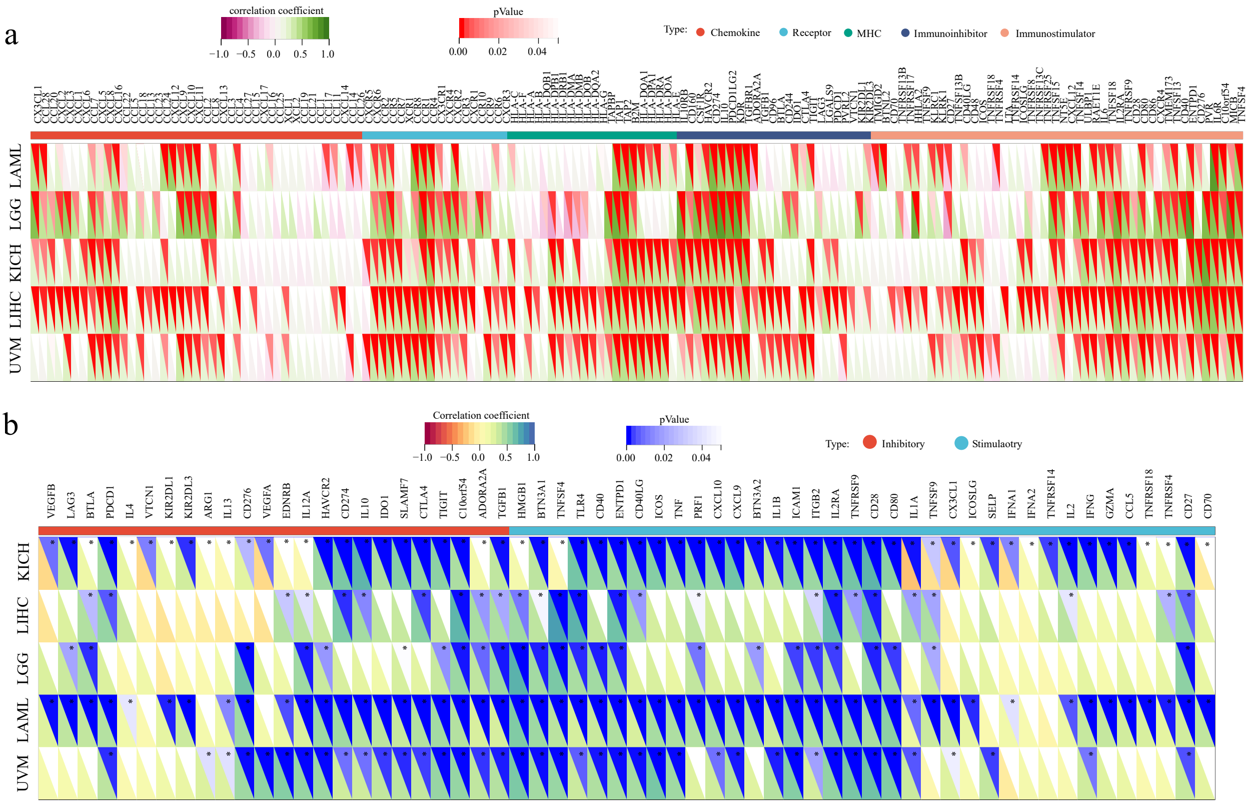

a
correlation coefficient
−1.0 −0.5 0.0 0.5 1.0
pValue
0.00 0.02 0.04
Type: Chemokine Receptor MHC Immunoinhibitor Immunostimulator
CX3CL1 CCL28 CCL20 CXCL2 CXCL3 CXCL1 CXCL6 CCL7 CXCL5 CXCL8 CXCL16 CCL22 CCL5 CCL18 CCL13 CCL23 CCL24 CXCL12 CXCL9 CXCL10 CXCL11 CCL2 CCL8 CXCL13 CCL3 CCL4 CCL27 CCL15 CXCL17 CCL16 CCL25 XCL1 XCL2 CCL19 CCL21 CCL1 CCL17 CCL11 CXCL14 CCL14 CCL26 CXCR5 CXCR6 CCR2 CCR5 CCR7 XCR1 CCR8 CCR1 CCR4 CX3CR1 CXCR4 CXCR2 CCR3 CXCR1 CCR10 CCR9 CCR6 CXCR3 HLA-C HLA-F HLA-A HLA-B HLA-DQB1 HLA-DPB1 HLA-DRB1 HLA-DMA HLA-DMB HLA-DOB HLA-DQA2 HLA-G TAPBP TAP1 TAP2 B2M HLA-DQA1 HLA-DPA1 HLA-DRA HLA-DOA HLA-E IL10RB CD160 CSF1R HAVCR2 CD274 IL10 PDCD1LG2 KDR TGFBR1 ADORA2A TGFB1 CD96 BTLA CD244 IDO1 CTLA4 TIGIT LAG3 LGALS9 PDCD1 PVRL2 VTCN1 KIR2DL1 KIR2DL3 TMIGD2 BTNL2 CD70 TNFRSF13B TNFRSF17 HHLA2 TNFSF9 KLRC1 KLRK1 CD27 TNFSF13B CD40LG CD48 ICOS TNFRSF18 TNFRSF4 LTA TNFRSF14 ICOSLG TNFRSF8 TNFRSF13C TNFRSF25 TNFSF15 NT5E CXCL12 TNFSF14 ULBP1 RAET1E IL6 TNFSF18 IL2RA TNFRSF9 CD28 CD80 CD86 CXCR4 TMEM173 TNFSF13 CD40 ENTPD1 CD276 PVR IL6R C10orf54 MICB TNFSF4
LAML
LGG
KICH
LIHC
UVM
b
Correlation coefficient
−1.0 −0.5 0.0 0.5 1.0
pValue
0.00 0.02 0.04
Type: Inhibitory Stimulaotry
VEGFB LAG3 BTLA PDCD1 IL4 VTCN1 KIR2DL1 KIR2DL3 ARG1 IL13 CD276 VEGFA EDNRB IL12A HAVCR2 CD274 IL10 IDO1 SLAMF7 CTLA4 TIGIT C10orf54 ADORA2A TGFB1 HMGB1 BTN3A1 TNFSF4 TLR4 CD40 ENTPD1 CD40LG ICOS TNF PRF1 CXCL10 CXCL9 BTN3A2 IL1B ICAM1 ITGB2 IL2RA TNFRSF9 CD28 CD80 IL1A TNFSF9 CX3CL1 ICOSLG SELP IFNA1 IFNA2 TNFRSF14 IL2 IFNG GZMA CCL5 TNFRSF18 TNFRSF4 CD27 CD70
KICH
LIHC
LGG
LAML
UVM
